# Prevalence of Overweight and Obesity Among Children and Adolescents With Autism Spectrum Disorder and Associated Risk Factors

**DOI:** 10.3389/fped.2019.00038

**Published:** 2019-02-20

**Authors:** Norazlin Kamal Nor, Azilawati Hanim Ghozali, Juriza Ismail

**Affiliations:** Department of Paediatrics, Faculty of Medicine, UKM Medical Center, The National University of Malaysia, Kuala Lumpur, Malaysia

**Keywords:** autism, overweight, obesity, sleep habits, physical activity, feeding problems

## Abstract

**Introduction:** Prevalence of obesity in Autism Spectrum Disorder (ASD) has been reported to be higher than in the general population. Determining prevalence may help increase awareness of obesity in ASD and potentially lead to initiatives to reduce obesity. In order to understand obesity in ASD children, common risk factors were assessed including physical activity, feeding problems and sleep disturbances.

**Methods:** This is a cross-sectional study performed at the Child Development Center at Universiti Kebangsaan Malaysia Medical Center on 151 ASD children aged 2–18 years. Anthropometric and demographic information were obtained and parents completed three questionnaires; Children Sleep Habits Questionnaire (CSHQ), Physical Activity for Older Children Questionnaire (PAQ-C) and Brief Autism Mealtime Behavior Questionnaire (BAMBI).

**Results:** For ASD children in our sample, the prevalence of overweight (BMI ≥85th to <95th percentiles) was 11.3% and the prevalence of obesity (BMI ≥95th percentile) was 21.9%. The overweight/obese ASD children's median age was higher at 8.5 years (IQR 5.81–10.13) compared to the normal/underweight group of 6.33 years (IQR 4.75–7.7) with a *p*-value of 0.001. The two groups also differed significantly for maternal BMI and paternal age. The median maternal BMI in the overweight/obese group was 26.05 (IQR 23.35–32.25), statistically significantly higher (*p* = 0.003) than in the non-overweight/obese group, 24.7 (IQR 21–27.9). The median paternal age of 40 years (IQR 37–44) was statistically significantly higher (*p* = 0.039) in the overweight/obese group, compared to the median paternal age in the non-overweight/obese group of 38 (IQR 35–42). The male overweight/obese children had median PAQ-C score of 2.44 (IQR 2.00–3.00) vs. 2.89 (IQR 2.35–3.53) in the counterpart group with a *p*-value of 0.01. Using the multiple linear regression stepwise method, three predictors associated with BMI percentiles reached a statistical level of significance; PAQ-C score in males (*p* < 0.001), the BAMBI domains of Food Refusal (*p* = 0.001) and Limited Variety of Food (*p* = 0.001).

**Conclusions:** The prevalence of obesity and overweight is high among Malaysian ASD children and adolescents. Older child age, high maternal BMI, older paternal age, low physical activity, low likelihood of food refusal and high likelihood of food selectivity were found to be risk factors for high BMI in these children.

## Introduction

The prevalence of childhood obesity is increasing rapidly in Malaysia in tandem with increasing global prevalence. In the U.S., 13.9% of children and adolescents aged 2–19 were obese in 1999 and the trend showed a significant increase to 17.1% between 2003 and 2004. The prevalence remains high, having been reported in a recent study to be approximately 17.2% (1). In 2004 in Malaysia, a study reported the prevalence of overweight and obesity among school children and adolescents to be 7.3% (2). Recent studies found that the prevalence of overweight and obesity among children in Malaysia have increased significantly to 18.2–19.9 and 15.2%, respectively (3, 4). Obesity has become a significant global public health problem with unhealthy excess weight posing health risks for chronic diseases such as Type 2 diabetes mellitus, hypertension, dyslipidaemia, orthopedic problems, and sleep disordered breathing (5–9). The study of childhood obesity in a country like Malaysia, in which children below 18 years make up approximately 30% of the population, may provide a good representation of the direction of obesity prevalence of children worldwide.

Interestingly, several studies have revealed that the prevalence of obesity is significantly greater among children with autism spectrum disorder (ASD), compared to the general population. Based on previous literature on ASD, the prevalence of obesity among ASD children ranged from 17 to 32%, and the prevalence of overweight ranged between 13 and 33% (10–18). A recent study in the U.S based on the 2016 National Survey of Children's Health reported that the prevalence of overweight and obesity are higher in the ASD group in comparison to typically developing children, with 19 and 23% of children with ASD reported to be overweight and obese, respectively, compared to 14 and 15% of typically developing children (18).

ASD is a neurodevelopmental disorder characterized by impairments in communication, behavior, and social functioning beginning in childhood. There are no local epidemiological studies on ASD prevalence in Malaysia. However, a feasibility study on the use of Modified Checklist for Autism in Toddlers (M-CHAT) among children aged 18–36 months by the Ministry of Health Malaysia found the prevalence of ASD to be approximately 1.6 in 1,000, significantly lower than those reported by recent worldwide ASD prevalence studies, and likely an underestimation (19). Autism prevalence has been showing an increasing trend over the past few decades.

There are several risk factors that might contribute to obesity among ASD children. A significant proportion of ASD children have been reported to have sleeping and feeding problems, and difficulty in engaging in physical activity (20–22). Children with ASD have been found to be less physically active and fit compared to typically developing children (20). Studies by McCoy et al. (23) and Healy et al. (24) compared physical activity and obesity among adolescents with and without ASD and found that the ASD group are more likely to be obese and less likely to engage in regular physical activity or sports (23, 24).

There is a clear association between short sleep duration and the risk of childhood obesity in the general population (25, 26). Children with ASD have poorer sleep quantity and quality compared to typically developing children and these issues do not appear to improve with age (21). They commonly have a shorter duration of sleep and problems with sleep tend to endure. Humphreys et al. (27) investigated longitudinal sleep patterns in children with ASD and found that sleep duration in children with ASD reduced from 30 months of age onwards and persisted until adolescence. Night-time sleep duration was shortened by later bedtimes and earlier waking times (27).

Children with ASD are frequently associated with picky eating and aversions to specific textures, colors, smells, temperatures, and brand names of foods. The degree of food selectivity appears to be an important indicator of acceptance or rejection of food and might be related to obesity. Children with ASD exhibit greater likelihood of food selectivity, eat a narrower range of foods, and have been frequently reported to have behavioral concerns during mealtime such as screaming or crying and food refusal (22, 28–30). A possible risk factor for obesity is the use of food as a primary reinforcer to calm these children down, which could also lead to obesity (31). Another possible mechanism leading to food selectivity or refusal in ASD children is “gut-brain” axis disruption leading to gastrointestinal disturbances, which may potentially be amenable to dietary manipulation or supplementary management (32).

Obesity is especially challenging to manage in children with ASD and therefore it is important to identify significant modifiable risk factors for potential remedial measures. The standard management for obesity in children and adolescents in Malaysia, and in general worldwide, is the reduction of energy intake by dietary modifications, increasing physical activity, and reducing physical inactivity. These are carried out using behavior modification techniques for modifying eating habits and improving activity patterns, and the involvement of the family in weight management (Malaysian Obesity Clinical Practice Guideline). Obtaining adequate night time sleep has also been proven to be associated with 40% lower prevalence of obesity (33).

Previous studies have focused on the prevalence rate of overweight and obesity among the ASD population. There is, however, a paucity of research on physical activity and its impact on body mass index among ASD children, with no previous study yet reported in Malaysia. To the best of our knowledge, there is little research to identify other possible risk factors apart from physical activity associated with overweight and obesity in children with ASD, specifically the impact of sleep habits and mealtime behavior. The aims of this study are to assess the prevalence of overweight and obesity among Malaysian ASD children and adolescents its associated risk factors, and to determine the relationship between the level of physical activity, sleep habits and mealtime behavior with the BMI status of Malaysian ASD children.

## Methodology

This study was carried out at an outpatient setting on 151 children and adolescents aged 2–18 years who were diagnosed with ASD based on DSM IV or DSM 5 criteria and were on follow up at the Child Development Center (CDC) UKMMC. Patients' records were reviewed to obtain the diagnosis, co-morbidities, age, and sex. Stadiometer and weighing scale were used to obtain height and weight. Measured values for weight and height were used to calculate the body mass index (BMI) and these were charted on growth charts to determine percentiles by gender and age. Centers for Disease Control and Prevention criteria for BMI were used to define overweight and obesity (≥85th to <95th percentiles and ≥95th percentiles, respectively). Parental height, weight, and BMI were also measured. Questionnaires were given to parents who had signed the consent form and the questionnaires were filled up while they were waiting for their appointment. Questionnaires used were Children Sleep Habit Questionnaire (CHSQ), Physical Activity for Older Children Questionnaire (PAQ-C), and Brief Autism Mealtime Behavior Inventory (BAMBI). Exclusion criteria were children who were on Ritalin and antipsychotics.

This is a cross sectional study. The sample size was calculated based on the formula n' = NZ^2^ P(1–P)/d^2^(N−1) + Z^2^ P(1–P) whereby N is the population size, Z is the statistic for a level of confidence, P is the expected proportion, and *d* is precision (34). The estimated number of ASD patients under CDC follow up is 300 children, Z value is 1.96 for the level of confidence interval 95%, the expected prevalence of overweight plus obesity based on previous studies is 0.5 and the precision is 5%. The calculated sample size was 165.

Ethical approval was obtained from the Research Ethics Committee UKM (Research code: FF-2018-247). This ethical approval allowed for us to collect data from human subjects in the form of questionnaires and anthropometric data, with confidentiality maintained for all study subjects. Each participant (parent) was provided with an explanation of the study, both in written and verbal forms, and requested to sign a consent form prior to collection of data.

## Questionnaires

### Children Sleep Habit Questionnaire (CSHQ)

CSHQ is a retrospective 34-item questionnaire, developed by Owens et al. (35) that has been used to examine sleep behavior in young children aged 4–12 years (35). It is not intended to diagnose specific sleep problems but instead was designed to screen for the most common sleep problems. It is based on parental report and contains 8 subscales: bedtime resistance, sleep onset delay, sleep duration, sleep anxiety, night waking, parasomnias, sleep disordered breathing, and daytime sleepiness. The answers are based on a Likert scale. Items are rated on a 3-point scale; “usually” if the sleep behavior occurred 5–7 times per week, “sometimes” for 2–4 times per week and “rarely” for 0–1 time per week. The response “usually” is scored as 3, “sometimes” scored as 2, and “rarely” scored as 1. A total score of over 41 indicates a pediatric sleep disturbance. The questionnaire had been translated into the Malay version by Firouzi et al. (36) and was pretested on a sample of 17 children. The Cronbach's alpha of the questionnaire was 0.895, which indicates high reliability (36).

### Physical Activity for Older Children Questionnaire (PAQ-C)

The PAQ-C is a self-administered, 7-day recall questionnaire developed by Kowalski et al. (37) to assess general levels of physical activity of children aged ~8–14 years old (37). It has 10 items, 9 of which are scored on a 5-point scale. The final PAQ-C activity summary score is the mean value from 1 to 5 for each of 9 items. A score of 1 indicates low physical activity whereas a score of 5 indicates high physical activity. Reliability and validity data have been proven by Kowalski et al. (37). This questionnaire has been translated into the Malay language and validation performed by Mohd Zaki et al. on 73 children aged 10–17 years old, which showed that the Malay version has good internal consistency with Cronbach alpha 0.75–0.77 (38). Based on a study by Voss et al. (39) on 7,226 children aged 10–15 years old to determine the cut-off point for PAQ-C score, it was found that PAQ-C scores of more than 2.9 for boys and more than 2.7 for girls indicated “sufficiently active” group vs. “low active” group (39).

### Brief Autism Mealtime Behavior Inventory (BAMBI)

BAMBI was developed by Lukens (40) to measure mealtime behavioral problems seen in autistic children aged between 3 and 11 years old. It is a parental report and consists of 18 items that are in three categories of eating behavior: limited variety of food, food refusal, and features of autism. Items were rated on a 5-point Likert scale, where “1” referred to never/rarely, “2” for seldom, “3” for occasionally, “4” for often and “5” for almost every meal. It has been validated by Lukens (40). DeMand et al. (41) explored the psychometric properties of BAMBI in 273 children with autism and derived a cut-off for BAMBI total score. The cut off total score of more than 34 indicates problematic feeders (41). Permission from Dr. Colleen Taylor Lukens to use and translate BAMBI has been obtained for this study. BAMBI has been translated into the Malay language using forward and backward translation methods. The original BAMBI was translated into the Malay language by a bilingual person. The Malay version of BAMBI was then back-translated into English by another translator who had not seen the original English version. The two different translators were fluent in both the Malay language and English. The two versions of translation were found to be comparable after being checked by two pediatricians. A pilot study was conducted using the Malay version of the BAMBI on 30 subjects for validation. The Cronbach's alpha was found to be 0.83, which indicates good reliability.

The median age was 6.75 years old. One hundred and thirty two were male (87.4%) and 19 were female (12.6%). Ethnic distribution were 99 Malays (65.6%), 41 Chinese (27.2%), 8 Indians (5.3%), and 3 of “other” ethnicity (2%). Nine children had a co-morbidity of attention deficit hyperactive disorder (ADHD) (6%), 11 had global developmental delay (GDD) (7.3%), 4 had intellectual disability (ID) (2.6%), and 8 of the children had other co-morbidities including dysmorphism, epilepsy, *beta*-thalassemia, atopic eczema and speech language impairment (SLI) (5.3%). One child was on a gluten-restriction diet.

### Statistical Analysis

Data analysis was carried out using Statistical Package for Social Sciences (SPSS) Version 23.0. To compare the median difference or percentage difference of the variables between obese, overweight, normal weight, and underweight groups, the Mann-Whitney *U* test was used for continuous data and Chi squared test for categorical data. The variables included age, ethnicity, co-morbidities, number of siblings, socioeconomic status, parental BMI, parental age, and parental education. The Spearman Correlation was applied to determine the correlation between the variables. Multiple Linear Regression analysis utilizing stepwise method was performed for the association between sleep habits, physical activity level and mealtime behavior and odds of being overweight and obese.

The data was analyzed between two groups of ASD children based on BMI, comparing those with BMI ≥85th percentile (overweight + obese, *n* = 50) to those with BMI <85th percentile (underweight + normal, *n* = 101), to determine associations between being overweight/obese and its risk factors. Normality skewness and kurtosis test was performed, and it showed that the data was skewed. Therefore, non-parametric tests were utilized.

## Results

Table 1 demonstrates the breakdown of ASD children by BMI category. The prevalence of overweight individuals in ASD children in this study was 11.3%, with 17 of the 151 children having BMI ≥85th to <95th percentile. The prevalence of obesity was 21.9% with 33 of the 151 children having BMI ≥95th percentile. The total prevalence of overweight and obesity in ASD children in the study was therefore 33.2%, with 50 of the children having BMI ≥85th percentile.

**Table 1:** Body Mass Index (BMI) status of 151 children with ASD.

**BMI status by percentiles**	***N***	**%**
Underweight (BMI <5th percentile)	11	7.3
Normal (BMI ≥5th to <85th percentiles)	90	59.6
Overweight (BMI ≥ 85th to <95th percentiles)	17	11.3
Obese (BMI ≥ 95th percentiles)	33	21.9

**Figure 1:**
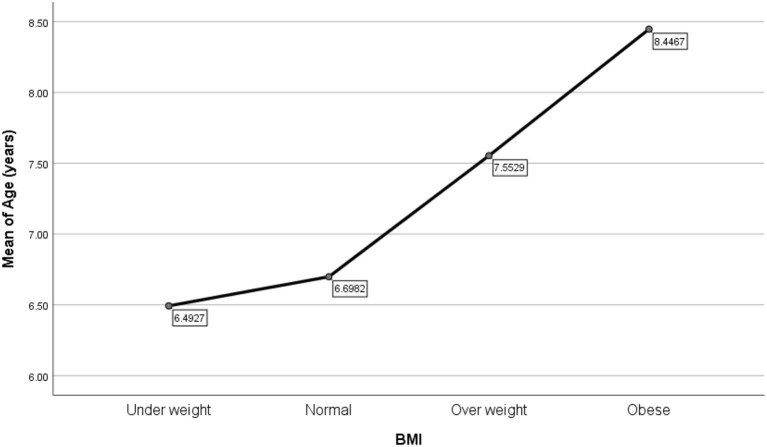
Weight status in relation to mean age (years).

Table 2 shows the characteristics of the study sample, comparing those categorized as overweight and obese to those who were not. There was a significant difference in median age between the two groups, whereby the overweight/obese ASD children's median age was 8.5 years (IQR 5.81–10.13), in comparison to non-overweight/obese group which was 6.33 years (IQR 4.75–7.7) with a *p*-value of 0.001. Figure 1 demonstrates the association between BMI percentiles and mean age, whereby increasing age is associated with higher BMI. The two groups also differed significantly in terms of maternal BMI and paternal age, whereby in the overweight/obese group, the maternal median BMI was statistically significantly higher than the non-overweight/obese group. The maternal median BMI in overweight/obese was 26.05 (IQR 23.35–32.25) and maternal BMI in non-overweight/obese was 24.7 (IQR 21–27.9).

**Table 2:** Comparison of characteristics of overweight and obese with normal and underweight groups.

**Characteristics**	**Normal + Underweight BMI <85th percentiles (*n* = 101)**	**Overweight + Obese BMI ≥ 85th percentiles (*n* = 50)**	***p*****-value**
	**Median (IQR) or n (%)**	**Median (IQR) or n (%)**	
Age (years)	6.33 (4.75–7.71)	8.54 (5.81–10.13)	0.001^*^
**SEX**			
Female	12 (11.9%)	7 (14%)	0.712
Male	89 (88.1%)	43 (86%)	
Birth weight (kg)	3.01 (2.73–3.32)	2.99 (2.70–3.20)	0.459
**RACE**			
Malay	69 (68.3%)	30 (60%)	0.252
Chinese	26 (25.7%)	15 (30%)	
Indian	4 (4%)	4 (8%)	
Others	2 (2%)	1 (2%)	
Paternal BMI	26.00 (23.6–29.00)	27.65 (24.43–31.25)	0.096
Maternal BMI	24.7 (21–27.9)	26.05 (23.35–32.25)	0.003^*^
Paternal age (years)	38 (35–42)	40 (37–44)	0.039^*^
Maternal age (years)	36 (34–39)	37 (34.75–40.25)	0.108
Monthly income (RM)	6,000 (4,000–10,000)	5,000 (3,000–10,000)	0.184
Number of siblings	2 (2–3)	2 (2–3)	0.393
**CO-MORBIDITIES**			
None	77 (76.2%)	42 (84%)	0.247
ADHD	7 (6.9%)	2 (4%)	
GDD	10 (9.9%)	1 (2%)	
ID	3 (3%)	1 (2%)	
Others	4 (4%)	4 (8%)	
**DIETARY RESTRICTION**			
None	97 (96%)	50 (100%)	0.514
Restriction	4 (4%)	0 (0%)	
**PATERNAL EDUCATION**			
Tertiary	73.5 (72.3%)	31 (62%)	0.644
Secondary	28 (27.7%)	19 (38%)	
**MATERNAL EDUCATION**			
Tertiary	75 (74.3)	31 (62%)	0.369
Secondary	26 (25.7%)	19 (38%)	

* *Significant median difference at p < 0.05 using Mann-Whitney U test/Significant number difference at p < 0.05 using Chi square test. ADHD, attention deficit hyperactive disorder; GDD, global developmental delay; ID, intelectual disability. The bold values are statistically significant at p < 0.05*.

Table 3 describes the frequency of sleep disturbance based on Total Sleep Disturbance Index, physical activity level and problematic feeders among the ASD children. All ASD children in this study (except two) have pediatric sleep disturbance. Parents of seven ASD children did not answer the PAQ-C questionnaire relating to physical activity as the questions were not applicable to their children, who at 3 years of age were too young. From a total of 144 children, 41.7% were sufficiently active whereas 53.6% had levels of activity that were low. From the feeding questionnaire, a majority (91.4%) of the children were problematic feeders.

**Table 3:** Frequency of sleep disturbance, physical activity level and problematic feeder among 151 children with ASD.

**Risk factors for Overweight/Obesity (Questionnaires)**		***N***	**%**
Pediatric sleep disturbance (CSHQ)	Yes	149	98.7
	No	2	1.3
Physical activity level (PAQ-C)	Sufficiently active	63	41.7
	Low active	81	53.6
	NA	7	4.6
Problematic feeder (BAMBI)	Yes	138	91.4
	No	13	8.6

In Table 4, obese and overweight ASD children were compared to ASD children who were underweight and had normal weight in relation to the studied risk factors. One hundred and forty nine out of 151 ASD children were identified as having disturbance with sleep based on Total Sleep Disturbance Index of the Children Sleep Habit Questionnaire (CSHQ). The median duration of sleep for the whole study sample was 9 h. Median sleep hours for overweight/obese group was 9 (IQR 8–10; *n* = 80) and for underweight/normal group the median sleep hours was 8.5 (7.63–9.88; *n* = 40). No significant difference was noted in Total Sleep Disturbance Index among the two groups. Stratifying by gender did not reveal a significant difference for Total Sleep Disturbance Index (*p* = 0.458). Thirty-one parents did not answer the total duration of sleep in hours.

**Table 4:** Comparison between normal and underweight ASD children with overweight and obese ASD children based on CSHQ, PAQ-C and BAMBI scoring.

	**Normal & Underweight (*n* = 101)**	**Overweight & Obese (*n* = 50)**	***p*****-value**
	**Median (IQR 25–75)**	**Median (IQR 25–75)**	
**i. CSHQ: TOTAL INDEX, TOTAL DURATION OF SLEEP AND EIGHT DOMAINS OF SLEEP HABIT**			
Total sleep disturbance index	54 (50.5–58)	55 (51–58)	0.909
Total duration of sleep (hours)	9 (8–10) (*n* = 80)	8.5 (7.63–9.88) (*n* = 40)	0.197
A. Bedtime resistance	12.5 (11–14)	12 (10–14)	0.414
B. Sleep onset delay	3 (2–3)	3 (2–3)	0.601
C. Sleep duration	3 (3–3)	3 (3–3)	1.000
D. Sleep anxiety	7 (5–8)	6 (5–8)	0.904
E. Night waking	4 (3–5)	3 (3–4)	0.081
F. Parasomnias	9 (7–10)	8 (7–10)	0.552
G. Sleep disordered breathing	3 (3–4)	3 (3–4.25)	0.124
H. Daytime sleepiness	14 (13–15.75)	14 (12.75–15)	0.608
**ii. PAQ-C**			
PAQC score			
Male	2.89 (2.35–3.53) (*n* = 84)	2.44 (2.00–3.00) (*n* = 42)	0.010^*^
Female	2.67 (2.14–3.27) (*n* = 12)	3.17 (2.81–3.75) (*n* = 6)	0.205
**iii. BAMBI AND DOMAINS**			
BAMBI total score	44 (40–48)	45 (42–50.5)	0.257
A. Limited variety of food	25 (22–27)	26 (23–28)	0.052
B. Food refusal	9 (7–11)	7 (5–9.25)	0.005^*^
C. Features of autism	11 (10–13)	12 (9.75–13.25)	0.196

* *Significant median difference at p < 0.05 using Mann-Whitney U test. CSHQ, children sleep habit questionnaire; PAQ-C, physical activity questionnaire for older children; BAMBI-brief autism mealtime behavior inventory. The bold values are statistically significant at p < 0.05*.

Based on gender stratification, there was a significant difference in the Physical Activity Questionnaire for Older Children (PAQ-C) score between the two groups amongst male ASD children. The male overweight/obese children had a median PAQ-C score of 2.44 (IQR 2.00–3.00) vs. 2.89 (IQR 2.35–3.53) in the counterpart group with a *p*-value of 0.01. The lower score indicates less physical activity. For the questionnaire on physical activity, seven of the children were too young for the questionnaire, resulting in assessment of 144 out of 151 children for this part of the analysis.

Of the 151 children in the study, 138 were problematic feeders based on BAMBI scoring. There was no significant difference between the two groups in the BAMBI score: however, one of the domains of BAMBI, the Food Refusal component, showed a significant difference between the two groups. The overweight/obese group had lower scores in the Food Refusal domain as compared to non-overweight/obese. The median score in overweight/obese group was 7 (IQR 5–9.25) and the median score in non-overweight/obese group was 9 (IQR 7–11). Gender stratification did not reveal a significant difference for total BAMBI score (*p* = 0.986).

Using multiple linear regression stepwise method, three predictors associated with BMI percentiles reached a statistical level of significance. These were the PAQ-C score in males (*p* < 0.001, beta coefficients −0.37) and the BAMBI domains of Food Refusal (*p* = 0.001, beta coefficients −0.71) and Limited Variety of Food (*p* = 0.001, beta coefficients 0.39). The beta coefficients for PAQ-C score in males and Food Refusal score showed a negative beta coefficients value, which indicates that they contribute to higher weight in ASD children. Whereas, Limited Variety of Food score has a positive beta coefficients value, which indicates that it is protective against higher weight status. Table 5 illustrates the strength of association between PAQ-C score for male ASD, and the two BAMBI domains mentioned previously. Based on the analysis, 74% of the change in BMI percentiles was significantly affected by three factors: Physical Activity Questionnaire for Older Children (PAQ-C) score, total scores for BAMBI domains of Food Refusal and Limited Variety of Food.

**Table 5:** Predictors of overweight/ obesity among children and adolescents with ASD.

**Risk factors**	**B**	**95% CI**	***p*****-value**
PAQ-C score for male ASD	−0.37	−2.95, −1.14	<0.001^*^
Total Food refusal score	−0.71	−1.01, −0.41	0.001^*^
Total Limited Variety of Food score	0.39	0.22, 0.57	0.001^*^

*R^2^ = 0.74 (adjusted R^2^ = 0.69)*,

* *significant p-value < 0.05*.

## Discussion

The prevalence of overweight and obesity in ASD children in our study were 21.9 and 11.3%, respectively. Our results for prevalence of obesity in ASD children are consistent with previous studies of obesity prevalence in ASD, although our study showed a lower prevalence of overweight individuals (10–18). The prevalence of overweight individuals in ASD children in our study was similar to that reported by Memari et al. (12). A possible hypothesis for this lower prevalence in overweight ASD children is that in our population, those who had difficulty maintaining an ideal weight were more severe in phenotype and thus more likely to present to a tertiary center such as ours. However, we did not assess for severity in this study and were not able to investigate this further. Similar to other published studies, our findings showed a higher prevalence of obesity in ASD children compared to what has been reported in children from the general population (11, 14). A recent study of Malaysian children from the general population found an obesity prevalence of 15% (4). Our study is timely as obesity is becoming a greater problem in children in general, especially in ASD children.

We found that the BMI status of ASD children change as they age. The younger age group tend to be underweight and the older age group tend to be obese. Hill et al. (17) found that the prevalence of overweight and obesity were significantly higher in the age groups of 2–5 years and 12–17 years (17). Our findings showed similar results in the older age group, although we did not find comparable results for the younger children. However, the majority of the children in our study were above 4 years of age and this may account for the dissimilar findings. Must et al. (42) also demonstrated that the prevalence of obesity in ASD children increased from the age of 10–17 years, consistent with the trend observed in our study. They also found that typically developing children who were obese became less obese with increasing age, whereas children with ASD did not (42). A study by MacDonald et al. (43) found that there is a decline in physical activity as children with ASD age, especially for moderate to vigorous physical activities (43). One possible explanation for this is that children with ASD may have challenges in engaging in physical activity due to motor and social skills, and communication difficulties, that result in more time spent sedentarily (44). A higher BMI in older ASD children may be the result of less physical activity as children with ASD age, which could be due to increasing difficulty for parents to manage older children with ASD.

We found that overweight and obesity among ASD children were associated with higher maternal BMI. In our study, the median maternal BMI in overweight/obese group was 26, which is classified as overweight. Some probable causes may be an unhealthy family diet and lack of family-centered exercise, both of which could result in mother and child concurrently having a higher weight. Paternal BMI in overweight/obese ASD children also showed an increasing trend, but did not reach statistical significance. A postulation for why maternal BMI was found to be associated with obesity in ASD children is that children are more likely to have a similar diet and activity level as their mothers, since in our setting mothers are the most probable primary caretaker. Overweight and obesity is also found to be associated with older paternal age. It is possible that older fathers may be less likely to engage children in physical activity, and thus these children spend more time sedentarily, potentially leading to increased risk of obesity. However, we did not specifically explore this in our study.

The majority (98.7%) of children in our study had sleep disturbance when scored on the Total Sleep Disturbance Index. Previous studies based on parental reports found that children with ASD are more likely to have disturbed sleep as compared to typically developing children, including short sleep duration, frequent night waking and long sleep onset latency (27, 45). ASD children with sensory over-responsivity may be particularly predisposed to sleep disturbance and hyperarousal (46). The median duration of sleep for all patients in this study was 9 h. Based on a meta-analysis conducted by Chen et al. (25), the recommended sleep duration for children age 5–10 years old is at least 10 h (25). This shows that the majority of ASD children in this study were not getting the recommended duration of sleep for their age. However, there was no significant difference in sleep habits and sleep duration between overweight/obese ASD children and their non-overweight/non-obese counterpart. Sleep disturbance is common in ASD, but our study did not show that it was a risk factor for obesity.

Analysis for level of physical activity was stratified based on gender. Stratification was done because the cut-off point for males and females were different. Girls require a lower score to be defined as sufficiently active compared to boys. Low physical activity level was found in male ASD children who were overweight and obese. This result is consistent with a previous study by McCoy et al. (23) which reported that adolescents with ASD were more likely to be overweight and obese and less likely to engage in regular physical activity (23). Reduction in activity has been recognized as a risk factor for obesity in children (47). This suggests that ASD children who have low levels of physical activity are at risk of overweight and obesity, and the mainstay of intervention for obesity in these children is greater participation in physical activity. In addition, healthcare practitioners should advocate prevention of obesity in ASD children by routinely enquiring about physical activity levels during clinical assessment and advising more physical activity, in order to reduce the risk of obesity. No significant difference was observed in physical activity level among girls with ASD, which may be due to the very small number of female patients.

The majority (91.4%) of subjects have problems with feeding. Previous studies have reported that ASD is associated with feeding problems (22, 28–30). Reasons for feeding difficulties may be related to core autistic features such as insistence on sameness and rigidity as well as sensory issues. Gastrointestinal disturbances have also been frequently reported among the ASD population and this could potentially affect food intake and food choice (32). There was no significant difference in the BAMBI score between the two groups. However, food refusal was observed more frequently in the non-overweight/non-obese group. This may reduce the likelihood of them being obese or overweight. There was less food refusal observed in those with high BMI. In our study we also found that higher BMI in ASD children was associated with food selectivity. Children with high BMI are thus more likely to choose only specific foods, but not refuse food in general. Some ASD children have been noted to have a diet with a narrow repertoire of food types, for example, a preference for crunchy foods, which is likely related to sensory-seeking behavior. Children with ASD who have high food selectivity may choose foods that are high in calories, leading to increased BMI. The results of our study are consistent with previous studies in which it has been demonstrated that ASD children are more likely to have feeding problems. However, further research is required to assess the possible mechanisms by which feeding problems may contribute to the development of obesity.

## Limitations

This study was only conducted on children with ASD. There was no data from typically developing children from the general population. As such, the prevalence rate in our study was compared to existing prevalence of obesity in children from the general population, based on studies conducted earlier.

The selection of subjects was not randomized. The 151 children in this study were taken as convenient sampling in the CDC during the period of data collection. Ideally, a larger sample size of 320, considering the effect size, is required to accurately represent the Malaysian ASD population in studies where subjects are not randomized. However, due to time constraints, the sample size obtained for this study was less than the calculated value to correct for precision. This may potentially impact the precision of the results. The value of precision that we obtained in our study using the sample size, was smaller, *d* = 0.02, which means that the statistical cut-off level should be less than *p* < 0.02. Having said that, almost all of our significant results had a *p*-value of less than 0.02. Therefore, the results of our analysis were still valid for most of our findings. The questionnaire used for physical activity was designed for children aged 8–14 years, but in this study, we utilized it to include younger children from 3 years and above, which is not ideal. PAQ-C questionnaire has been previously used and validated in Malaysia.

This study combined normal and underweight children which may affect analysis, however because the underweight group is small, we feel it is unlikely to affect the overall result.

CSHQ is a screening tool for sleep disturbance in children from the general population. As children with ASD have been reported to have a greater likelihood of sleep difficulties, it is not surprising that the vast majority of our children were found to have sleep disturbance. However, no specific questionnaire has been developed to ascertain sleep disturbance in ASD children.

## Conclusion

From this study, the prevalence of overweight and obesity is high among Malaysian ASD children and adolescents, at 21.9 and 11.3%, respectively. Older age group, high maternal BMI, older paternal age, low physical activity, low likelihood of food refusal and higher likelihood of food selectivity, were all found to be risk factors for high BMI in these children. Clinicians who manage children with ASD need to have a greater awareness of the increased risk of overweight and obesity in ASD so that they can provide meaningful preventive measures regarding obesity.

## Data Availability

Datasets are available on request: The raw data supporting the conclusions of this manuscript will be made available by the authors, without undue reservation, to any qualified researcher.

## Author Contributions

NK and JI contributed to the concept and design of the study. AG and NK organized the database and performed the statistical analysis. NK, AG, and JI drafted the manuscript. All authors contributed to manuscript revision, read, and approved the submitted version.

### Conflict of Interest Statement

The authors declare that the research was conducted in the absence of any commercial or financial relationships that could be construed as a potential conflict of interest.

## References

[1] Ogden CL Carroll MD Fryar CD Flegal KM Prevalence of obesity among adults and youth: United States, 2011–2014. NCHS Data Brief (2015) 219:1–8. 26633046

[2] Moy FM Gan CY Zaleha MK. Body mass status of school children and adolescents in Kuala Lumpur, Malaysia. Asia Pac J Clin Nutr. (2004) 13:324–9. 15563435

[3] Naidu BM Mahmud SZ Ambak R Sallehuddin SM Mutalip HA Saari R. Overweight among primary school-age children in Malaysia. Asia Pac J Clin Nutr. (2013):22:408–15. 10.6133/apjcn.2013.22.3.18 23945411

[4] Lee PY Cheah WL Chang CT Siti Raudzah G. Childhood obesity, self-esteem and health-related quality of life among urban primary schools children in Kuching, Sarawak, Malaysia. Mal J Nutr. (2012) 18:207–19. 24575667

[5] Goran MI Ball GDC Cruz ML. Obesity and risk of type 2 diabetes and cardiovascular disease in children and adolescents. J Clin Endocrinol Metab. (2003) 88:1417–27. 10.1210/jc.2002-021442 12679416

[6] Friedemann C Heneghan C Mahtani K Thompson M Perera R Ward AM. Cardiovascular disease risk in healthy children and its association with body mass index: systematic review and meta-analysis. BMJ (2012) 345:e4759. 10.1136/bmj.e4759 23015032 PMC3458230

[7] Freedman DS Mei Z Srinivasan SR Berenson GS Dietz WH. Cardiovascular risk factors and excess adiposity among overweight children and adolescents: the Bogalusa Heart Study. J Pediatr. (2007) 150:12–7.e2. 10.1016/j.jpeds.2006.08.042 17188605

[8] Taylor ED Theim KR Mirch MC. Orthopedic complications of overweight in children and adolescents. Pediatrics (2006) 117:2167–74. 10.1542/peds.2005-1832 16740861 PMC1863007

[9] Bixler EO Vgontzas AN Lin HM. Sleep disordered breathing in children in a general population sample: prevalence and risk factors. Sleep (2009) 32:731–6. 10.1093/sleep/32.6.731 19544748 PMC2690559

[10] Chen AY Kim SE Houtrow AJ Newacheck PW. Prevalence of obesity among children with chronic conditions. Obesity (2009) 18:210–3. 10.1038/oby.2009.185 19521350

[11] Curtin C Anderson SE Must A Bandini L. The prevalence of obesity in children with autism: a secondary data analysis using nationally representative data from the National Survey of Children's Health. BMC Pediatrics (2010) 10:11. 10.1186/1471-2431-10-11 20178579 PMC2843677

[12] Memari AH Kordi R Ziaee V Mirfazeli FS Setoodeh MS Weight status in Iranian children with autism spectrum disorders: investigation of underweight, overweight and obesity. Res Autism Spectrum Disord. (2012) 6:234–9. 10.1016/j.rasd.2011.05.004

[13] Egan AM Dreyer ML Odar CC Beckwith M Garrison CB. Obesity in young children with autism spectrum disorders: prevalence and associated factors. Childhood Obes. (2013) 9:125–31. 10.1089/chi.2012.0028 23485020

[14] Bicer AH Alsaffar AA. Body mass index, dietary intake and feeding problems of Turkish children with autism spectrum disorder (ASD). Res Dev Disabil. (2013) 34:3978–87. 10.1016/j.ridd.2013.08.024 24029808

[15] Broder-Fingert S Brazauskas K Lindgren K Iannuzzi D Van Cleave J. Prevalence of overweight and obesity in a large clinical sample of children with autism. Acad Pediatr. (2014) 14:408–14. 10.1016/j.acap.2014.04.004 24976353

[16] Zuckerman KE Hill AP Guion K Voltolina L Fombonne E. Overweight and obesity: prevalence and correlates in a large clinical sample of children with autism spectrum disorder. J Autism Dev Disord. (2014) 44:1708–19. 10.1007/s10803-014-2050-9 24488158 PMC4058357

[17] Hill AP Zuckerman KE Fombonne E. Obesity and autism. Pediatrics (2015) 136:1051–61. 10.1542/peds.2015-1437 26527551 PMC4657601

[18] Healy S Aigner CJ Haegele JA. Prevalence of overweight and obesity among US youth with autism spectrum disorder. Autism (2018). 10.1177/1362361318791817. [Epub ahead of print]. 30101597

[19] Family Health Division Prosiding Mesyuarat Membincangkan Hasil Kajian Saringan dan Pengendalian Masalah Autisme. Kuala Lumpur: Malaysian Health Technology Assessment Section (MaHTAS), Ministry of Health Malaysia, Federal Government of Malaysia (2006).

[20] Tyler K MacDonald M Menear K. Physical activity and physical fitness of school-aged children and youth with autism spectrum disorders. Autism Res Treat. (2014) 2014:312163. 10.1155/2014/312163 25309753 PMC4182001

[21] Hodge D Carollo TM Lewin M Hoffman CD Sweeney DP. Sleep patterns in children with and without autism spectrum disorders: developmental comparisons. Res Dev Disabil. (2014) 35:1631–8. 10.1016/j.ridd.2014.03.037 24780146

[22] Schreck KA Williams K Smith AF. A comparison of eating behaviors between children with and without autism. J Autism Dev Disord. (2004) 34:433–8. 10.1023/B:JADD.0000037419.78531.86 15449518

[23] McCoy SM Jakicic JM Gibbs BB. Comparison of obesity, physical activity, and sedentary behaviors between adolescents with autism spectrum disorders and without. J Autism Dev Disord. (2016) 46:2317–26. 10.1007/s10803-016-2762-0 26936162

[24] Healy S Haegele JA Grenier M Garcia JM. Physical activity, screen-time behavior, and obesity among 13-year olds in Ireland with and without autism spectrum disorder. J Autism Dev Disord. (2016) 47:49–57. 10.1007/s10803-016-2920-4 27671801

[25] Chen X Beydoun MA Wang Y. Is sleep duration associated with childhood obesity? A systematic review and meta-analysis. Obesity (2008) 16:265–74. 10.1038/oby.2007.63 18239632

[26] Caputi P Iverson D. Lack of sleep could increase obesity in children and too much television could be partly to blame. Acta Paediatr. (2014) 103:e27–31. 10.1111/apa.12447 24117519

[27] Humphreys JS Gringras P Blair PS Scott N Henderson J Fleming PJ. Sleep patterns in children with autistic spectrum disorders: a prospective cohort study. Arch Dis Child (2013) 99:114–8. 10.1136/archdischild-2013-304083 24061777 PMC3913218

[28] Bandini LG Anderson SE Curtin C Cermak S Evans EW Scampini R. Food selectivity in children with autism spectrum disorders and typically developing children. J Pediatr. (2010) 157:259–64. 10.1016/j.jpeds.2010.02.013 20362301 PMC2936505

[29] Evans EW Must A Anderson SE Curtin C Scampini R Maslin M. Dietary patterns and body mass index in children with autism and typically developing children. Res Autism Spectr Disord. (2012) 6:399–405. 10.1016/j.rasd.2011.06.014 22936951 PMC3427936

[30] Attlee A Kassem H Hashim M Obaid RS. Physical status and feeding behavior of children with autism. Indian J Pediatr. (2015) 82:682–7. 10.1007/s12098-015-1696-4 25663296

[31] Kral TVE Souders MC Tompkins VH Remiker AM Eriksen WT Pinto-Martin JA. Child eating behaviors and caregiver feeding practices in children with autism spectrum disorders. Public Health Nurs. 32:488–97. 10.1111/phn.12146 25112438

[32] Santocchi E Guiducci L Fulceri F Billeci L Buzzigoli E Apicella F. Gut to brain interaction in autism spectrum disorders: a randomized controlled trial on the role of probiotics on clinical, biochemical and neurophysiological parameters. BMC Psychiatry (2016) 16:183. 10.1186/s12888-016-0887-5 27260271 PMC4893248

[33] Anderson SE Whitaker RC. Household routines and obesity in US preschool-aged children. Pediatrics (2010) 125:420–8. 10.1542/peds.2009-0417 20142280

[34] Naing L Winn T Rusli BN Practical issues in calculating the sample size for prevalence studies. Arch Orofacial Sci. (2006) 1:9–14.

[35] Owens JA Spirito A Mc Guinn M. The Children's Sleep Habits Questionnaire (CSHQ): psychometric properties of a survey instrument for school-aged children. Sleep (2000) 23:1043–51. 10.1093/sleep/23.8.1d 11145319

[36] Firouzi S Poh BK Ismail MN Sadeghilar A. Sleep habits, food intake, and physical activity levels in normal and overweight and obese Malaysian children. Obes Res Clin Prac. (2014) 8:e70–8. 10.1016/j.orcp.2012.12.001 24548579

[37] Kowalski KC Crocker PRE Faulkner RA Validation of the physical activity questionnaire for older children. Pediatr Exer Sci. (1997) 9:174–86. 10.1123/pes.9.2.174

[38] Mohd Zaki NA Sahril N Omar MA Ahmad MH Baharudin A Mohd Nor NS Reliability and validity of the physical activity questionnaire for older children (PAQ-C) in Malay language. Int J Public Health Res. (2016) 6:670–6.

[39] Voss C Ogunleye AA Sandercock GR. Physical activity questionnaire for children and adolescents: English norms and cut-off points. Pediatr Int. (2013) 55:498–507. 10.1111/ped.12092 23461812

[40] Lukens CT Linscheid TR. Development and validation of an inventory to assess mealtime behavior problems in children with autism. J Autism Dev Disord. (2008) 38:342–52. 10.1007/s10803-007-0401-5 17578658

[41] DeMand A Johnson C Foldes E. Psychometric properties of the brief autism mealtime behaviors inventory. J Autism Dev Disord. (2015) 45:2667–73. 10.1007/s10803-015-2435-4 25813517 PMC4554795

[42] Must A Eliasziw M Phillips SM Curtin C Kral TVE Segal M. The effect of age on the prevalence of obesity among US youth with autism spectrum disorder. Childh Obes. (2016) 13:25–35. 10.1089/chi.2016.0079 27704874 PMC5278796

[43] MacDonald M Esposito P Ulrich D. The physical activity patterns of children with autism. BMC Res Notes (2011) 4:422. 10.1186/1756-0500-4-422 22008607 PMC3213672

[44] Finlay J Bejerot S Hanley M. Motor deficits in children with autism spectrum disorder: a cross-syndrome study. Autism Res. (2014) 7:664–76. 10.1002/aur.1408 25258309

[45] Wiggs L Stores G. Sleep patterns and sleep disorders in children with autistic spectrum disorders: insights using parent report and actigraphy. Dev Med Child Neurol. (2004) 46:372–80. 10.1017/S0012162204000611 15174528

[46] Mazurek MO Petroski GF. Sleep problems in children with autism spectrum disorder: examining the contributions of sensory over-responsivity and anxiety. Sleep Med. (2015) 16:270–9. 10.1016/j.sleep.2014.11.006 25600781

[47] Steinbeck KS. The importance of physical activity in the prevention of overweight and obesity in childhood: a review and an opinion. Obes Rev. (2001) 2:117–30. 10.1046/j.1467-789x.2001.00033.x 12119663

